# Social network addiction symptoms and body dissatisfaction in young women: exploring the mediating role of awareness of appearance pressure and internalization of the thin ideal

**DOI:** 10.1186/s40337-022-00643-5

**Published:** 2022-08-08

**Authors:** Rafael Delgado-Rodríguez, Rocío Linares, María Moreno-Padilla

**Affiliations:** Department of Psychology, University of Jaén, Campus Las Lagunillas, 23009 Jaén, Spain

**Keywords:** Body dissatisfaction, Social network site addiction, Awareness of appearance-based pressures, Internalization of thin ideal, Body mass index

## Abstract

**Background:**

Previous studies testing for a direct relationship between social networking sites (SNS) addiction and body dissatisfaction (BD) have yielded inconsistent results. Here, we aimed to identify underlying processes that could mediate this relationship. Specifically, we studied the relationship between SNS addiction symptoms and BD through the awareness of appearance pressures and the internalization of beauty ideals, both individually and serially: SNS addiction → Awareness → BD; SNS addiction → Internalization → BD; SNS addiction → Awareness → Internalization → BD.

**Method:**

A total of 368 female undergraduates with SNS accounts completed scales to assess SNS addiction symptoms (Social Network Addiction Questionnaire), BD (Body Shape Questionnaire), awareness, and internalization (Sociocultural Attitudes Towards Appearance Questionnaire-4; awareness and internalization scales). A theoretical serial mediation model was constructed to examine the proposed relationships. Body mass index was included as a covariate to control the influence of this important variable.

**Results:**

The results indicated that both awareness and internalization independently mediated the relationship between SNS addiction symptoms and BD. Also, there was a significant serial mediation effect; women with more SNS addiction symptoms tended to be more aware of appearance pressure, which was associated with the internalization of beauty ideals. In turn, this internalization was positively related to BD symptoms.

**Conclusions:**

These findings shed light on the indirect relationship between SNS addiction and BD, demonstrating independent and accumulative mediating effects of awareness and internalization.

**Supplementary Information:**

The online version contains supplementary material available at 10.1186/s40337-022-00643-5.

## Plain English summary

Is social network site (SNS) addiction directly associated with body dissatisfaction (BD)? Previous studies examining a direct relationship between these variables have reported inconsistent results. We investigated whether other variables mediate this relationship (i.e., variables that are affected by SNS addiction and, in turn, provoke BD). Specifically, we examined whether being aware of the pressures to achieve beauty ideals, and internalization thereof (hereafter referred to as awareness and internalization, respectively) mediate the SNS addiction–BD relationship, both individually and serially: SNS addiction → Awareness → BD; SNS addiction → Internalization → BD; and SNS addiction → Awareness → Internalization → BD. 368 female undergraduates completed scales assessing SNS addiction symptoms, BD, awareness, and internalization. Mediational analyses indicated that SNS addiction symptoms predict greater awareness and internalization, moreover, both variables were positively related to women’s BD. Further, the findings indicated that awareness and internalization were serial mediators of the relationship between SNS addiction and BD; women with more severe SNS addiction symptoms were more aware of appearance-related pressure, which was associated with the internalization of beauty ideals. In turn, this internalization was positively related to BD symptoms. These results indicate an indirect link between SNS addiction and BD, highlighting the role of awareness and internalization as mediators of this relationship.

**Supplementary Information:**

The online version contains supplementary material available at 10.1186/s40337-022-00643-5.

## Background

Body image can be defined as the attitudinal disposition toward one’s own physical appearance, which includes evaluative, cognitive and behavioral components [1]. If the evaluation of body image becomes negative, i.e., there is discrepancy between one’s actual and ideal body, individuals are at risk of becoming unhappy with their appearance, which is considered as body dissatisfaction (BD) [2, 3]. BD intensifies rapidly in late adolescence and is extremely widespread among college-age women [4–6], which is notable given its role as a risk factor for eating disorder symptoms [7, 8]. Among the etiological factors of BD, repeated exposure to beauty ideals has been extensively studied [9]. The channels through which exposure to these ideals occurs have changed according to new forms of communication [10].

Before the advent of social networking sites (SNSs), the traditional mass media (e.g., TV commercials and programs, magazines, etc.) consumed in Western societies were the most pervasive and powerful influence [11, 12], exerting negative effects on body image [13–18]. Currently, SNSs and specially appearance-based SNSs (i.e., those involving appearance-oriented activities such as the posting and viewing of photos) are recognized as problematic in terms of body image and eating behaviors [19–21], given the importance assigned to physical appearance [22, 23]; SNSs users selectively self-present their—and are presented with—most attractive and idealized body photos [24–26].

Notwithstanding the negative effect of SNSs on body image, they are used widely in Western societies, especially among young adults [27]. The high accessibility of SNSs (through smartphones) and variety of services offered may provoke excessive and compulsive use of these applications, which can lead to SNS addiction [28–30]. Although the concept of SNS addiction is currently controversial [31], some authors stressed that SNS overuse might cause dependency [32] and consider it as a form of addiction [29, 30, 33, 34] comparable to other behavioral addictions and substance use disorders [35–37]; the overall prevalence of SNS addiction is 5% (according to studies using a monothetic or strict monothetic classification; [38]). Individuals with SNS addiction are preoccupied with, and strongly motivated to use, SNSs. Moreover, contrary to persons with a “healthy” high SNS use pattern (i.e., for whom time spent on SNSs has no negative effect; [39]), the excessive time and effort devoted to SNSs in individuals with SNS addiction negatively impacts their other social activities, studies/job, and/or psychological well-being [33].

Individuals with symptoms of SNS addiction connect more frequently to SNSs associated with appearance-related activities (e.g., Instagram, Facebook, Snapchat, and Twitter), and spend more time on those platforms [40–42]; furthermore, a greater proportion of their time is spent on viewing profiles (vs. non-addictive users; [43]). These aspects are notable because they have also been related to greater BD [21, 44]. Moreover, SNS addiction symptoms have been associated with other factors implicated in BD [42], such as lower self-esteem [45], greater desire for a thinner body [46], the number of methods used to change their body [47], the perception of one’s own body as being fatter than it actually is [48], and disordered eating behaviors [49]. However, the few studies that aimed to directly link SNS addiction with BD reported contradictory results. While one study found a relationship between Instagram addiction symptoms and BD [50], several other studies did not find that addiction to Instagram, or SNSs in general, was directly linked to BD [40, 51, 52]. Based on studies that identified mediating variables between SNS use and BD (for a review, see 20), we consider that underlying processes could be mediating the relationship between SNS addiction symptoms and BD. To the best of our knowledge, previous studies have not examined such indirect link. Identifying these underlying mechanisms might shed light on the contradictory results of previous studies that examined the SNS addiction-BD connection, and help us understand how SNS addiction impacts SNS users’ body image, which can have clinical implications (e.g., by informing preventive programs aiming to decrease the impact of SNS overuse on BD).

Regarding key mediating variables, some authors have highlighted the role of awareness of appearance-related pressures; i.e., being aware of beauty social standards and perceiving external pressure to achieve them [8, 11]. Media messages, peers, and family are the three primary sources of social pressure on women to be thin [53]. Currently, appearance-based SNSs are an important route through which idealized female body types are transmitted to the public [54], given that these SNSs–such as Facebook–have a major impact on body consciousness [55]. For example, previous literature indicated that more frequent SNS use predicted more frequent reception of appearance-related feedback from peers [44]. Likewise, SNS trends such as fitspiration and thinspiration (which represent forms of social pressure [56] and are promulgated on platforms such as Instagram, Facebook, YouTube, and Twitter) are associated with greater BD [20, 57]. According to these results, we hypothesize that women with more severe SNS addiction will show greater appearance-related awareness, which can be associated with more severe symptoms of BD (H1: SNS addiction → Awareness → BD).

The internalization of beauty ideals (i.e., when individuals cognitively “buy into” socially prescribed appearance ideals and engage in behaviors aimed to approximate and achieve them) has also been highlighted as a mediating variable in the association of BD with exposure to traditional and Internet media [9, 58–60]. In the context of SNSs, internalization of beauty ideals has been shown to be associated with SNS use [21, 60–62] and mediates the association between Facebook engagement or the time spent on this SNS and BD [55, 63]. Accordingly, we hypothesize that women with SNS addiction symptoms might show higher levels of internalization of beauty standards, which can be related to higher BD (H2: SNS addiction → Internalization → BD).

Notably, a synergistic effect of awareness and internalization on BD has been established. The tripartite model highlights internalization of beauty ideals as a channel whereby pressures to be thin (from peers, family, and the mass media) affects individuals’ BD [53], and in part through this variable (i.e., BD), internalization is also related to disordered eating. The mediational role of internalization between awareness and BD has considerable empirical support (e.g., [8, 53, 64–67]; for a review see [9]). In fact, some studies considered internalization of the thin ideal as a necessary condition for the sociocultural pressures to adhere to the thin ideal to lead to substantive BD [58, 59]. Considering that SNSs (especially, appearance-based ones) have a major impact on body consciousness [55], because they are a key route through which idealized female body types are transmitted to the public [54], and that SNS addiction symptoms are associated with having more SNS accounts, connect more frequently and spend more time on appearance-based SNSs [42], we might expect SNS addiction symptoms to be related to BD through concurrent awareness and internalization (H3: SNS addiction → Awareness → Internalization → BD).

**Fig. 1:**
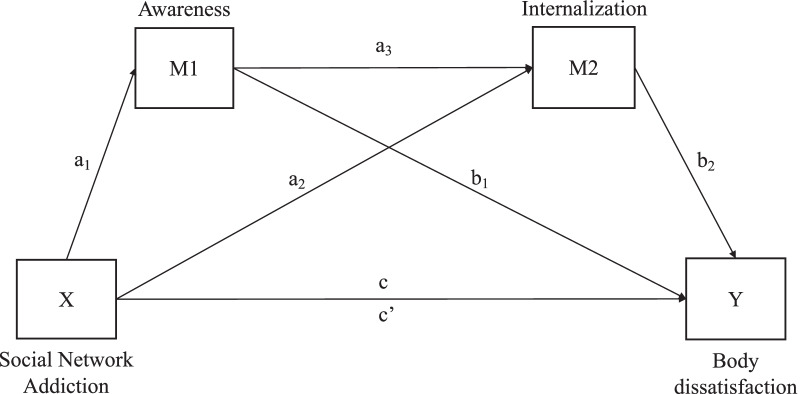
The proposed serial mediation model

The current study aims to examine the indirect relationship between SNS addiction symptoms and BD in female undergraduates, by exploring the mediational role of awareness and internalization both independently (i.e., H1 and H2) and concurrently (i.e., H3). To this end, we constructed a serial mediational model between SNS addiction symptoms and BD (using the Body Shape Questionnaire [BSQ]; [68]), including awareness of appearance-related pressures and internalization of the thin ideal (using the Sociocultural Attitudes Towards Appearance Questionnaire-4 [SATAQ-4] awareness and internalization subscales; [69]) as mediating variables. Given the variety of SNSs used by college students, most of whom also use several SNSs (e.g., the majority of participants in Linares et al. [70] used four or five SNSs), a scale to assess addiction to a specific SNS (e.g., the Bergen Facebook Addiction Scale; [29]) would have not been representative of all potential participants. Therefore, we used the Social Network Addiction scale (SNA; [71]) as a global measure of SNS addiction symptoms. Body mass index was included in the model as a covariate to control its influence, given its role in predicting female BD [72]. Based on previous literature, we hypothesize that women with SNS addiction symptoms (who are frequently exposed to beauty standards due to their greater use of appearance-based SNSs; [40]) will show greater awareness and internalization of thin ideals, which will be positively associated with greater BD, both individually and serially. The hypothesized model is depicted in Fig. 1.

## Methods

### Participants

A total of 387 undergraduate women from the University of Jaén (Andalusia, Spain) participated voluntarily in this study, receiving course credits as compensation. We excluded participants who provided incorrect answers to catch questions (“What is 2 + 2?”, “Have you ever had a fatal heart attack?”; n = 9), provided implausible data (e.g., 2020 as birth year; n = 2), or were aged above 30 years (n = 6). To ensure homogeneity in social network use patterns, participants whose mobile phones were broken or not used for accessing social networks were excluded (n = 2). The final sample was composed of 368 women ranging in age from 17.8 to 30 years (M = 20.54 years, SD = 2.22). This research was conducted according to the Declaration of Helsinki and the Ethics Committee of the University of Jaén approved this study (Nov.20/2.PRY).

### Measures

#### Social media use-related questions

Several questions were used to characterize social media use (Table 1). First, the participants listed all of the SNSs that they use. They also reported how much time they spend on SNSs during weekdays and on the weekend on an 8-point Likert scale (1, < 10 min/day; 2, 10–30 min/day; 3, 30–60 min/day; 4, 1–2 h/day; 5, 2–3 h/day; 6, 3–5 h/day; 7, 5–10 h/day; 8, > 10 h/day). Furthermore, they indicated the average time that they spent on Facebook and Instagram in the last week (this information is provided by both of these SNSs). The frequency of connection to SNSs was indicated via a 6-point Likert scale (1, Connected all the time; 2, 7–12 times/day; 3, 3–6 times/day; 4, 1–2 times/day; 5, 2–3 times/week; 6, 1 time/week). The participants reported the number of pictures submitted per week on a 5-point Likert scale (1, < 2; 2, 2–4; 3, 4–6; 4, 6–8; 5, > 8) and the frequency of submission of pictures to SNSs on a scale ranging from 1("never”) to 5 ("always”). Finally, the women answered questions related to photo-editing (of pictures of themselves, posted online or shared via mobile); these questions were taken from the Photo Manipulation Scale [73], which was translated into Spanish.

**Table 1:** Sociodemographic and SNS use-related question data

Questions	M (SD)
Age	20.54 (2.22)
BMI	22.46 (3.89)
Number of SNSs	3.97 (1.44)
Time on SNSs, weekday	4.33 (1.10)
Time on SNSs, weekend	4.59 (1.29)
Frequency of connection to SNSs	4.84 (0.81)
Frequency of submission of pictures to SNSs	3.26 (0.87)
Number of pictures submitted per week	1.48 (0.82)
Mean time spent on Facebook per day over the past 7 days	7.41 (27.08)
Mean time spent on Instagram per day over the past 7 days	97.56 (89.17)
Self-photo-editing behaviors	19.86 (5.90)

BMI, body mass index; SNSs, social network sites; M, mean; SD, standard deviation

#### Social network addiction questionnaire (SNA) [71]

This scale, which is based on the DSM-IV-TR criteria for the diagnosis of substance addiction [74], comprises 24 items scored using a 5-point Likert-type scale ranging from 0 (“never”) to 4 (“always”). This instrument is designed to measure addiction to social media in university students (e.g., "I feel anxious when I cannot connect to social media"). The original SNA has three factors (obsession, lack of personal control, and excessive use); however, in a recent study, the developers of the SNA argued that it has better psychometric properties if used as an unidimensional scale [75]. The total score ranges from 0 to 96, where higher scores indicate more severe social network addiction. The SNA does not have a cut-off point for diagnosis; however, a previous study that used latent profile analyses to group participants according to SNA scores distinguished between different groups of SNS addiction severity (and where 11% of Spanish college students were classified into the “high addiction” group) [76]. The SNA exceeded the critical values proposed in the literature as reliability indices, and the fit indices were in line with reports from the scientific community. The total internal consistency of the scale for our sample was 0.93.

#### Body shape questionnaire (BSQ) [77]

This instrument, consisting of 34 Likert-type items, assesses dissatisfaction with weight or shape and general preoccupation with, and distress about, body shape and size. Items are answered using a 6-point Likert-type scale ranging from 1 (“never”) to 6 (“always”). The total score ranges from 34 to 204, and higher scores indicate greater dissatisfaction. We used the Spanish version of the BSQ [68], for which adequate reliability and validity were reported when applied to undergraduate women [78]. The total internal consistency of the scale for our sample was 0.97.

#### Sociocultural attitudes towards appearance questionnaire-4 (SATAQ-4) [79]

This scale was used to determine the degree of assimilation of Western cultural standards of appearance. It constitutes 22 items scored using a 5-point Likert-type scale ranging from 1 (“definitely disagree”) to 5 (“definitely agree”). The SATAQ-4 can be divided into two subscales: awareness and internalization. The awareness scale comprises 12 items that measure the respondent perceives pressure from family, peers, and media to look thin or athletic. The internalization scale consists of 10 items that evaluate acceptance or self-imposed pressure to look thin or athletic. The total score ranges from 12 to 60 for the awareness scale and from 10 to 50 for the internalization scale, with higher scores indicating greater awareness and internalization, respectively. We used the Spanish version of the SATAQ-4 [69], which has shown excellent internal consistency when applied to college students. In our sample, the total internal consistency was 0.88 for the awareness scale and 0.91 for the internalization scale.

### Procedure

The study was performed online. We presented the research to participants in their lecture classrooms, and those who showed interest in participating were invited in groups (mean of 50 individuals per group) to attend a live Google Meet session. Participants were online throughout the whole task to maximize control of their environment during the experiment. They were instructed to avoid distractions (this was emphasized several times before the experiment). After obtaining informed consent, participants were presented with a definition of social network, with the aim of ensuring homogeneity in terms of understanding; social network was defined as a web-based service that allow individuals to (1) construct a public or semipublic profile within a bounded system, (2) generate a list of other users with whom they share connections, and (3) view and explore their list of connections, and those of other users within the system [80]. Afterwards, they answered sociodemographic and social network use-related questions (Table 1), and completed a short battery of questionnaires (BSQ, SATAQ-4, and SNA; see *Measures* section). We generated four pseudorandomized orders of questionnaires to control for order effects. At the end of the experiment, the participants were thanked and debriefed.

### Data analysis

Descriptive analyses were performed to explore the distribution of the data. Scores more than 3 SD above or below the mean for a given variable were winsorized to render the data distributions less sensitive to outliers while maintaining statistical power; a small proportion of variables were winsorized (SNA global score: 0.27% [*n* = 1]; body mass index: 1.63% [*n* = 6]). We then performed Pearson’s bivariate correlation analysis of the variables of interest (i.e., SNS addiction, BD, awareness, and internalization). Afterwards, a serial multiple mediation model was tested using model 6 of the PROCESS v3.5 macro for SPSS (ver. 21.0; SPSS Inc., Chicago, IL, USA) [81]. In this model, SNS addiction was the X variable, BD was the Y variable, and awareness and internalization were serial mediators (M1 and M2, respectively) (Fig. 1). Given that higher body mass index is associated with greater BD, we included it as a covariate [72]. The indirect effects of SNS addiction on BD were tested using a bootstrap estimation approach with 10,000 samples, and estimates were made at a 95% bootstrap confidence interval using the percentile method. Confidence intervals not containing zero were considered significant [82]. Indirect effect sizes were computed both as completely standardized effects [83] and indirect-to-direct-effect ratio statistics (P_M;_ [84]).

## Results

Means, standard deviations, normality estimates, and Pearson’s bivariate correlations between variables are shown in Table 2. Pearson’s bivariate correlation results indicated that BD was positively associated with all variables. Likewise, SNS addiction, awareness, and internalization were significantly related to one another. The intercorrelations among the variables provides initial support for the hypothesized indirect effects.

**Table 2:** Descriptive statistics, normality estimates and Pearson correlation coefficients between the research variables

	M	SD	Skewness (SE)	Kurtosis (SE)	1	2	3	4	5
Variables									
1 SNA	35.96	14.65	.23 (.13)	.01 (.25)	−				
2 Awareness	26.69	9.56	.34 (.13)	− .49 (.25)	.22^**^	−			
3 Internalization	26.41	8.78	− .11 (.13)	− .77 (.25)	.20^**^	.48^**^	−		
4 BSQ	88.56	35.02	.63 (.13)	− .32 (.25)	.20^**^	.65^**^	.65^**^	−	
5 BMI	22.46	3.89	1.13 (.13)	1.23 (.25)	− .14^**^	.38^**^	.14^**^	.36^**^	−

M, mean; SD, standard deviation; SE, standard error; SNA, social network addiction; BMI, body mass index; BSQ, body shape questionnaire

**p < .01

Table 3 shows the results of the serial multiple mediation model, in which awareness and internalization mediate the relationship between SNS addiction and BD (Fig. 1). The results showed that SNS addiction predicted awareness (a1, *p* < 0.001) and internalization (a2, *p* < 0.05). Awareness was also found to have a positive effect on internalization (a3, *p* < 0.001). Both awareness (b1, *p* < 0.001) and internalization (b2, *p* < 0.001) had a significant impact on BD. Moreover, the total effect (c, *p* < 0.001) of SNS addiction on BD was significant. However, the (direct) effect of SNS addiction on BD (c’, *p* = 0.123) was not significant after controlling for the impact of the other variables.

**Table 3:** The serial multiple mediation model, in which awareness and internalization mediate the relationship between social network addiction and body dissatisfaction

Path	Effect	SE	LLCI	ULCI		
Total effect (c)	.6078***	.1132	.3851	.8304		
Direct effect (c′)	.1340	.0850	− .0332	.3012		
a1	.1784***	.0305	.1185	.2383		
a2	.0591*	.0289	.0022	.1160		
a3	.4276***	.0475	.3341	.5210		
b1	1.3294***	.1536	1.0273	1.6314		
b2	1.7466***	.1532	1.4454	2.0479		

Indirect effects			BootLLCI	BootULCI	Std. effect	P_M_
Total indirect effects	.4737	.0774	.3261	.6285	.1982	0.7794
Indirect 1	.2372	.0456	.1528	.3311	.0992	0.3903
Indirect 2	.1033	.0524	.0041	.2061	.0432	0.1700
Indirect 3	.1332	.0285	.0835	.1939	.0557	0.2192

SE, standard error; LLCI, lower limit confidence interval; ULCI, upper limit confidence interval; Effect, unstandardized regression coefficient. BootLLCI, bootstrapping lower limit confidence interval; BootULCI, bootstrapping upper limit confidence interval; Std. effect, completely standardized indirect effects; P_M_, the ration of the indirect effect to the total effect. Indirect 1, social network addiction → awareness → body dissatisfaction; Indirect 2, social network addiction → internalization → body dissatisfaction; Indirect 3, social network addiction → awareness → internalization → body dissatisfaction

*p < .05

***p < .001

All three hypothesized mediating effects are supported. Both awareness (Indirect 1) and internalization (Indirect 2) mediate the association between SNS addiction and BD. It was also found that SNS addiction promoted BD through awareness and internalization concurrently (Indirect 3; i.e., serial mediation effect). With respect to the size of indirect effects, overall, the P_M_ statistics indicated that indirect effects accounted for 78% of the total effect of SNA addiction on BD.

## Discussion

Using a serial mediation model, the current study examined the indirect relationship between SNS addiction symptoms and BD through the awareness of appearance pressures and internalization of beauty standards. Body mass index was included as a covariate, given its role in predicting female BD [72]. Confirming our hypotheses, the findings indicate that women with more severe SNS addiction symptoms showed greater awareness and internalization, which independently were associated with more severe symptoms of BD (H1: SNS addiction → Awareness → BD; H2: SNS addiction → Internalization → BD). Moreover, SNS addiction symptoms were associated with BD, mediated synergistically by awareness and internalization (H3: SNS addiction → Awareness → Internalization → BD). Finally, our results support experimental research that did not find a direct influence of SNS addiction on BD [40, 51, 52]. Overall, the results of current study help to overcome inconsistences in the literature regarding the relationship between addictive SNS use and BD, highlighting the importance of considering mediating processes.

We found that SNS addiction symptoms were indirectly associated with BD through awareness. The first part of the mediation relationship (i.e., path a1) indicates that women with more severe SNS addiction are more aware of pressures to achieve beauty standards. This is in line with previous literature that considered appearance-based SNSs as powerful means of transmitting body ideals [54]; viewing attractive individuals receiving positive body-related comments might reinforce beauty ideals, thereby increasing appearance awareness and pressures to meet beauty standards [85]. The second part of the SNS addiction-BD mediation relationship (i.e., path b1) indicates that women with higher awareness suffer from more BD. Similarly, previous studies indicated that BD is increased by certain activities (e.g., viewing and commenting on photos or “body talk” [i.e., interpersonal virtual interactions focused on bodies]) and trends on SNSs (e.g., fitspiration/thinspiration) that might enhance awareness of the social pressure to be thin [20, 21, 56, 57, 86]. Overall, previous data indicate that young female undergraduates with symptoms of SNS addiction have higher awareness of appearance pressure, which is associated with greater dissatisfaction with their bodies.

This study demonstrates that internalization of the thin ideal is another mediating variable whereby addictive SNS use influences BD. Specifically, women scoring higher for SNS addiction showed greater internalization of the thin ideal (i.e., path a2), in line with studies indicating that more SNS use is associated with greater assimilation of appearance ideals as personal standards, i.e., internalization [61, 87, 88]. As our results show (i.e., path b2), adopting these unrealistic ideals as personal standards is related to BD symptoms, given that women tend to compare themselves against unrealistic body images [89]. In turn, these findings suggest that internalization of the thin ideal could mediate the relationship between addictive SNS use and BD, which is concordant with studies highlighting internalization as the mechanisms whereby SNS use (e.g., frequency and duration of use of Facebook, photo-related activities on Instagram) affects BD [55, 63, 87, 90].

We argue that the women with more severe SNS addiction symptoms in our sample likely scored higher for awareness and internalization due to more frequent exposure to appearance-related content. In line with this, and in accordance with previous studies [40, 41], more severe symptoms of SNS addiction are significantly predicted by indexes related to higher engagement on SNSs that focus on physical appearance, such as time spent on Instagram per week, the number of pictures submitted per week, and self-photo-editing behaviors (see Additional file 1). Moreover, 98% (n = 361) of our cohort used Instagram and/or Facebook, which are both SNSs known to be associated with pressure to be thin [21, 56].

A considerable body of literature stresses that awareness and internalization synergistically promote body disturbances; internalization of the thin ideal seems to be a key process whereby awareness can provoke BD symptoms [9, 53, 58, 59, 64–67, 91]. Our results support this previous finding in the context of SNSs; we found that the SNS addiction-BD relationship was serially mediated by awareness and internalization. In other words, women with more severe SNS addiction symptoms who are aware of appearance pressures are more likely to develop BD due to internalization of the thin ideal. Among studies addressing the mediating role of internalization of the thin ideal, some have indicated that this internalization is a necessary condition for appearance pressure to promote BD (e.g., [58, 59]); however, our results do not support this. Instead, our findings indicate that awareness of pressure to be thin (enhanced by SNS addiction) is sufficient to produce BD.

Previous studies examining the direct impact of SNS addiction on body concerns yielded contradictory results. While one study found a significant link between Instagram addiction symptoms and BD [50], others did not find that addiction to Instagram or SNSs in general was directly associated with BD [40, 51, 52]. Our results are in line with the latter group of studies, in that we did not find a direct effect of SNS addiction symptoms on BD (while controlling for body mass index), highlighting that SNS addiction does not negatively affect body image per se*,* but only through certain underlying processes such as awareness and internalization.

In line with previous research showing that body mass index is robustly associated with BD (e.g., [92, 93]), we found a significant correlation between both variables. Moreover, body mass index significantly correlated (in Pearson correlation analyses) with the other variables in the model. In order to control for this important variable, body mass index was included in the mediation model as a covariate. Further examination of the effect of body mass index in the serial multiple mediation model showed that this variable significantly predicted awareness (B_unstandardized coefficient_ = 1.0295; *p* < 0.001) and BD (B_unstandardized coefficient_ = 1.5395; *p* < 0.001), but not internalization (B_unstandardized coefficient_ = -0.0545; *p* = 0.636). Therefore, in the current model, higher body mass index (which was included as a covariate) was associated, in addition with higher BD, with greater awareness of appearance pressure, in line with previous literature (e.g., individuals with overweight status are more likely to report diet/muscle-related dialogue than underweight individuals; [94]). However, the current model indicated that body mass index was not associated with internalization of thin ideal, in line with previous research positing that women with a high body mass index may reject societal norms and not wish to conform to the thin ideal [95].

The findings of the current study should be evaluated while considering several limitations. First, the study was cross-sectional, where longitudinal research is more suitable to test mediation processes. Although our results have significant relevance in terms of our understanding of the associations between addictive SNS use and BD, those associations could be examined in future longitudinal and experimental studies investigating the causal mechanisms of BD. Another drawback is that our sample consisted only of college-age female Spanish participants, which might reduce the external validity and generalizability of the results. In order to overcome this limitation, samples with different socio-demographic characteristics could be enrolled in future studies. Ultimately, it should be noted that many studies examining the effect of SNS use on body concerns focused on mere exposure to those platforms, and did not consider other aspects of addictive SNS use (e.g., associating the frequency of SNS use with body image concerns [44]). However, we assessed the –indirect– link between symptoms of aberrant SNS use (e.g., obsessive and compulsive use of these platforms) and BD. In this sense, the current results should be interpreted with caution; they should be understood within the context of addictive use of SNSs, and might not be necessarily generalized to regular users of SNSs who show a healthy SNS use.

Notwithstanding these limitations, our study is significant in that it has theoretical implications for understanding the relationship between the addictive SNS use and body concerns. We showed the importance of considering the underlying mechanisms mediating the SNS addiction-BD link (contrary to previous studies that examined the direct relationship between these variables). Our findings indicate that addictive use of SNS plays an important role in the tripartite model, which stipulates that appearance pressure (from three sources; family, peers, and traditional media) lead to internalization of a thin appearance ideal, resulting in BD [53]. In this sense, women with addictive SNS use are more likely to be aware of appearance pressure (i.e., SNS addiction impacts on body consciousness); if such awareness arises in women who have internalized beauty ideals, they are more likely to show BD symptoms. The manner by which SNS addiction impacts body consciousness might be explained because these individuals are more engaged in appearance-oriented activities (e.g., self-photo-editing behaviors [96] or viewing profiles [43]) and spend more time on appearance-based SNSs [42]; which are a known source of appearance pressure [44].

The identification of those mechanisms responsible for women with addictive SNS use being more vulnerable to BD also has clinical implications. Prevention programs at the high school and university levels focusing on norms and policies that promote healthy online social networking should incorporate strategies to prevent body-related problems, providing students with strategies to deal with appearance-related pressures, and make it clear that SNSs portray unrealistic body images through applications that alter photographs; i.e., they depict idealized versions of the self. Psycho-educational interventions could focus on fostering a critical attitude toward the beauty ideals presented on SNSs. Strategies to reduce SNS use, such as using *apps* to cut down on the time spent on SNSs, could reduce compulsive use [97, 98] and therefore decrease exposure to body ideals. Likewise, identifying and decreasing the motivation for use of social media that negatively affects the intensity of SNS use and BD (e.g., using SNSs as a form of escapism from everyday life) could help prevent both problems [99]. In the field of body-related problems, psychologists should consider the effects of SNSs on patients’ psychological well-being, and view SNSs as sources of appearance pressure along with the three traditional sources (peers, family, and the traditional mass media). Developing strategies to decrease the impact of factors that contribute to BD has clear health implications that go beyond improving subjective evaluations of one's own body, given that BD is strongly associated with the development and maintenance of eating disorders [100–102]. BD has been shown to prospectively predict both restrained eating and bulimic symptoms [91, 103], moreover, it has been found to be associated with overestimation of weight and shape as determinants of self-worth, which represents the core psychopathological feature of eating disorders [104].

## Conclusions

Our findings contribute to the existing literature by demonstrating that, in the context of addictive SNS use, awareness of appearance-related pressures and internalization of beauty ideals exert both independent and synergistic (in a serial fashion) effects on young women, making them more vulnerable to BD. By contributing to theoretical understanding of the processes underlying individual differences in vulnerability to BD development in the context of SNS addiction, our findings represent a step forward in terms of clarifying the linkage between these variables.

## Supplementary Information

**Additional file 1**. Extra analyses to examine the influence of engagement with appearance-based social network sites on social network site addiction.

## Data Availability

The datasets used and/or analysed during the current study are available from the corresponding author on reasonable request.

## Abbreviations

BD: Body dissatisfaction
BMI: Body mass index
BSQ: Body Shape Questionnaire
BootULCI: Bootstrapping upper limit confidence interval
BootLLCI: Bootstrapping lower limit confidence interval
LLCI: Lower limit confidence interval
M: Mean
SATAQ-4: Sociocultural Attitudes Towards Appearance Questionnaire-4
SD: Standard deviation
SE: Standard error
SNA: Social networks addiction
SNS: Social networking sites
Std. effect: Completely standardized indirect effects
ULCI: Upper limit confidence interval
