# Supplementary material for: Social network addiction symptoms and body dissatisfaction in young women: exploring the mediating role of awareness of appearance pressure and internalization of the thin ideal
Source: J Eat Disord. 2022 Aug 8;10:117. doi: 10.1186/s40337-022-00643-5 (PMC9361684; doi:10.1186/s40337-022-00643-5)
Supplement: Supplementary file 1 — Additional file 1. Extra analyses to examine the influence of engagement with appearance-based social network sites on social network site addiction. [file 40337_2022_643_MOESM1_ESM.docx]

# Social network addiction symptoms and body dissatisfaction in young women: Exploring the mediating role of awareness of appearance pressure and internalization of the thin ideal

Rafael Delgado-Rodríguez, Rocío Linares, María Moreno-Padilla.

## Additional File 1

### Influence of engagement with appearance-based social network sites on social network site addiction

To explore if social network site (SNS) addiction symptoms are related to more frequent exposure to appearance-related content on SNSs, we examined the relationship between problematic SNS use (using the Social Network Addiction Scale [SNA]; Escurra Mayaute & Blas, 2014) and potential indices of higher engagement on SNSs that focus on physical appearance; i.e., time spent on Facebook and Instagram (appearance-based SNSs), submission of pictures of oneself to SNSs, self-photo-editing behaviors, and frequency of connection to SNSs. The latter variable was included because the vast majority of the sample (98 %) used Instagram and/or Facebook, then, frequency of connection to SNSs might be indicating the frequency of Instagram and/or Facebook use. To explore those relationships, we used a stepwise multiple linear regression analysis. Scores greater or less than 3 *SD* were winsorized to reduce the effect of outliers: a small proportion of variables was winsorized (time spent on Facebook and Instagram: 0.3% [n=1] and 2.2 % (n=8), respectively; submission of own pictures: 4.1% (n=15); self-photo-editing behaviors: 0.8% [n=3]; frequency of connection to SNSs: 0.3% [n=1]). The model explained 29% of the variance in SNS addiction symptoms. Problematic use of SNSs was predicted by the frequency of connection, self-photo-editing behaviors, time spent on Instagram and the number of pictures submitted. Positive correlation coefficients indicate that women with more severe SNS addiction symptoms scored higher on indices indicating greater engagement with appearance-based SNSs.

| **Table S1**  *Stepwise multiple linear regression to identify predictors of SNS addiction symptoms (global score).* | | | | | |
| --- | --- | --- | --- | --- | --- |
| Model | Predictors | B [95% CI] | Beta (SD) | R partial | Adj. R^2^ |
| 1 | Frequency of connection to SNSs | 0.37 [5.05 , 8.59] | 6.82 (0.90) | 0.37 | 0.13^***^ |
| 2 | Frequency of connection to SNSs | 0.35 [4.80 , 8.12] | 6.46 (0.84) | 0.37 | 0.24^***^ |
|  | Self-photo-editing behaviors | 0.34 [0.62 , 1.07] | 0.84 (0.11) | 0.36 |  |
| 3 | Frequency of connection to SNSs | 0.31 [3.99 , 7.33] | 5.66 (0.85) | 0.33 | 0.27^***^ |
|  | Self-photo-editing behaviors | 0.31 [0.55 , 0.99] | 0.77 (0.11) | 0.34 |  |
|  | Time spent on Instagram per week | 0.19 [0.02 , 0.06] | 0.04 (0.01) | 0.21 |  |
| 4 | Frequency of connection to SNSs | 0.28 [3.50 , 6.87] | 5.18 (0.86) | 0.30 | 0.29^***^ |
|  | Self-photo-editing behaviors | 0.29 [0.50 , 0.95] | 0.72 (0.11) | 0.32 |  |
|  | Time spent on Instagram per week | 0.18 [0.02 , 0.06] | 0.04 (0.10) | 0.20 |  |
|  | Number of pictures submitted per week | 0.14 [0.89 , 4.37] | 2.63 (0.89) | 0.15 |  |
| *Note:* B= unstandardized coefficient; Beta = standardized coefficient; Adj. R^2^ = adjusted R^2^; Δ_1_ = 0.11 for Model 2; Δ_2_ = 0.03 for Model 3; Δ_2_ = 0.02 for Model 4.  ^*^p < 0.05, ^**^p < 0.01, ^***^p < 0.001. | | | | | |

## References

Escurra Mayaute, M., & Salas Blas, E. (2014). Construcción y validación del cuestionario de adicción a redes sociales (ARS). *Liberabit*, *20*(1), 73-91. https://www.redalyc.org/pdf/686/68631260007.pdf
